# Empagliflozin-associated postoperative mixed metabolic acidosis. Case report and review of pathogenesis

**DOI:** 10.1186/s12902-023-01339-w

**Published:** 2023-04-14

**Authors:** Michal Sitina, Marek Lukes, Vladimir Sramek

**Affiliations:** 1 Department of anesthesiology and intensive care medicine, St. Anne´s University Hospital, Pekarska 664/53, Brno, 656 91 Czech Republic; 2 Department of Biostatistics, International Clinical Research Center, St. Anne´s University Hospital, Pekarska 664/53, Brno, 656 91 Czech Republic; 3 Faculty of Medicine, Masaryk University, Kamenice 5, Brno, 625 00 Czech Republic

**Keywords:** Empagliflozin, SGLT2 inhibitor, Euglycemic ketoacidosis, Hyperchloremic acidosis, Case report

## Abstract

**Background:**

Euglycemic diabetic ketoacidosis associated with SGLT2 inhibitors is a rare, relatively new and potentially fatal clinical entity, characterized by metabolic acidosis with normal or only moderately elevated glycemia. The mechanisms are not fully understood but involve increased ketogenesis and complex renal metabolic dysfunction, resulting in both ketoacidosis and hyperchloremic acidosis. We report a rare case of fatal empagliflozin-associated acidosis with profound hyperchloremia and review its pathogenesis.

**Case presentation:**

A patient with type 2 diabetes mellitus treated with empagliflozin underwent an elective hip replacement surgery. Since day 4 after surgery, he felt generally unwell, leading to cardiac arrest on the day 5. Empagliflozin-associated euglycemic diabetic ketoacidosis with severe hyperchloremic acidosis was identified as the cause of the cardiac arrest.

**Conclusions:**

This unique case documents the possibility of severe SGLT2 inhibitor-associated mixed metabolic acidosis with a predominant hyperchloremic component. Awareness of this possibility and a high index of suspicion are crucial for correct and early diagnosis.

## Background

Sodium-Glucose Transport Protein 2 Inhibitors (SGLT2i), called gliflozins, were originally developed for the treatment of type 2 diabetes mellitus (T2DM). However, their impressive cardiovascular and renal protective properties were later discovered [1–4]. Today, they are indicated in patients with heart failure regardless of ejection fraction and in patients with chronic kidney disease regardless of the presence of diabetes, [5–7] making them widely used drugs.

A rare but serious complication of SGLT2i use is euglycemic diabetic ketoacidosis [8] (eDKA). As shown in recent large prospective randomized trials, [9] its absolute risk is very low and does not outweigh the protective properties of SGLT2i when used properly. Safety has also been demonstrated in acute patients with COVID-19 [10]. However, in rare individual cases, this complication can be life-threatening, with a fatality rate of up to 1.5% [11].

We report a case of fatal mixed metabolic acidosis following elective hip replacement surgery in a patient treated with SGLT2i empagliflozin. This case represents a unique demonstration of the significant differences between classic DKA and SGLT2i-related ketoacidosis, which are rarely described in detail in the literature. We also discuss the mechanisms by which even mild euglycemic ketoacidosis can lead to the development of life-threatening hyperchloremic acidosis. As the use of SGLT2i becomes more widespread, it is likely that physicians will increasingly encounter this novel presentation of acidosis, which is still poorly understood in the medical community.

## Case presentation

A 61-year-old man with mild obesity, treated for T2DM with empagliflozin 10 mg/d, metformin 2000 mg/d and recently 12 units/day of long-acting insulin, underwent elective hip replacement surgery. Pre-operative assessment was unremarkable, with a low estimated risk of anesthesia (ASA 2). No oral antidiabetic agents were administered the evening before surgery. The surgery performed on day 1 (D1) was uneventful, as was the stay in the HDU. The administration of empagliflozin, metformin and insulin in chronic doses was resumed on D2. The patient was transferred to a ward on D3. On D4, the patient felt unwell and vomited once, but his condition improved after administration of metoclopramide. The attending physician revealed no remarkable findings. Blood gas analysis was not performed. On D5, the patient still felt unwell but without any specific complaints such as chest pain or dyspnea. Glycemia was below 10 mmol/l. In the evening, the patient vomited again. One hour later he suddenly collapsed due to cardiac arrest by ventricular fibrillation. CPR was started immediately, with ROSC after 35 min. Post-arrest ECG and echocardiography suggested acute myocardial infarction. Immediate coronary angiography revealed several significant chronic coronary artery stenoses but no acute culprit lesion, consistent with only minimal troponin T elevations over the following days. The left ventricle ejection fraction was 30–35% and there were no echocardiography findings suggestive of pulmonary embolism. On admission to the ICU, the patient was anuric and required norepinephrine. He died on D8 due to severe post-hypoxic brain injury.

On admission to the ICU one hour after CPR, a severe mixed metabolic acidosis was present with pH of 6.85 and base excess of -29 mmol/l (Table 1). Lactate was only moderately elevated (5.8 mmol/l). An elevated beta-hydroxybutyrate concentration of 3.2 mmol/l. Corresponded to a mild ketoacidosis [12]. Together with lactate and acetoacetate, whose concentration is about three times lower than that of beta-hydroxybutyrate, [13] the total organic acid concentration was about 10 mmol/l. The anion gap was 18.1 mmol/l, i.e. 9.6 mmol/l higher than expected [14]. Thus, the increase in the anion gap can be explained entirely by organic acids, with no other component. Subtracting this increase in anion gap still leaves a base excess of almost − 20 mmol/l, which must be explained by a non-anion gap hyperchloremic acidosis (admission Cl^−^ was 117 mmol/l, see Table 1). Overall, we found a mixed metabolic acidosis - lactic acidosis, ketoacidosis and hyperchloremic acidosis. We consider the lactic acidosis to be a consequence of cardiac arrest, but the other components of the acidosis, with base excess of -23 mmol/L after subtraction of the lactate, must have been present before CPR. We found no etiological explanation for the acidosis other than the SGLT2i-associated ketoacidosis. We hypothesize that SGLT2i gradually induced severe acidosis within 5 days of surgery, which triggered a malignant arrhythmia in the context of previously undiagnosed coronary artery disease. Here, we briefly review the pathogenesis of SGLT2i-associated acidosis and discuss some specific points of the presented case.

**Table 1:** Temporal development of acid-base parameters and electrolytes

						hours after CPR						Sampani et al. [22]
		day 0	day 2			1	4	8	22	32		
pH						6.85	6.82	6.94	7.27	7.26		7.05
pCO2	kPa					2.97	8.27	5.9	3.56	3.23		1.58
pO2	kPa					11.7	10.6	10	15.8	16.1		
Base excess	mmol/l					-29	-24	-22	-13	-15		
Bicarbonate	mmol/l					5.9	7.7	9	14.4	13.4		3
Lactate	mmol/l					5.8	3.1	2.3	1.4	1.4		0.6
Beta-hydroxybutyrate	mmol/l					3.2				0.3		
Sodium	mmol/l	137	139			141	140	137	141	142		133
Potassium	mmol/l	4.2	4.7			4.6	5	4.7	3.7	4.2		3.8
Chloride	mmol/l	103	108			117	113	112	115	119		113
Anion gap	mmol/l					18.1	19.3	16	11.6	9.6		16.9
Glucose	mmol/l	9.2	15.6			14.5	20.2	26	12.4	9.3		6.7
Haemoglobin	g/l	143	125			133	124	134	113	116		
Urea	mmol/l	4	7					11		17		
Creatinine	µmol/l	45	92					168		343		

Normal value of beta-hydroxybutyrate is under 0.5 mmol/l

With the albumin concentration of 35 g/l, the expected normal anion gap is 8.5 mmol/l [14].

## Discussion and conclusions

**Fig. 1:**
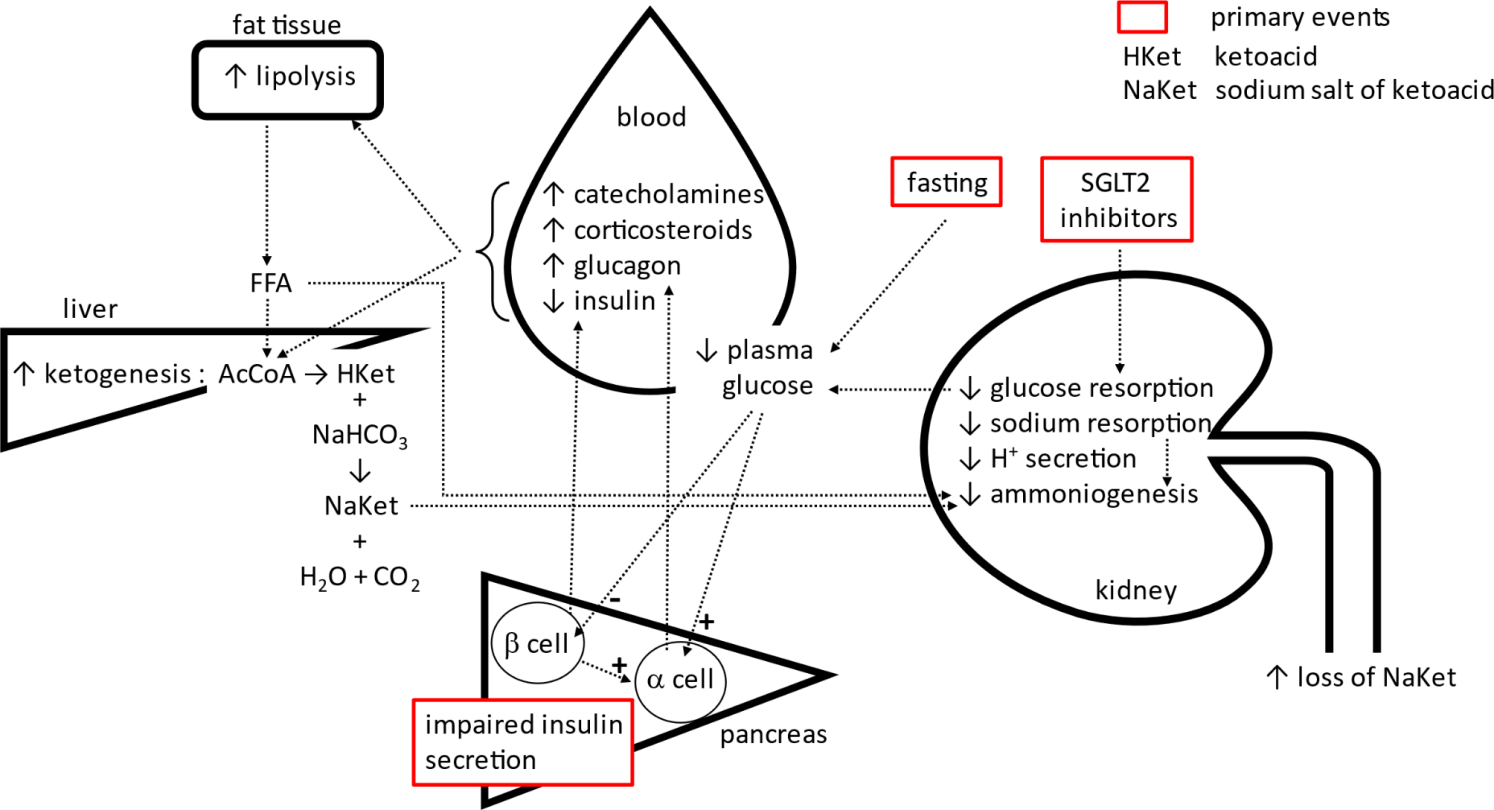
Pathogenesis of SGLT2 inhibitor–associated acidosis

SGLT2i block the SGLT2 co-transporter of sodium and glucose in the proximal tubule of the kidney, thereby reducing glucose reabsorption [15]. This seemingly simple mechanism of action results in a new, somewhat unstable metabolic equilibrium with increased ketone bodies formation and their renal elimination [16]. Precipitating factors such as surgery, acute illness, starvation or insulin deficiency can trigger highly complex metabolic and renal processes that can result in life-threatening metabolic acidosis (Fig. 1). Among the most important are increased ketogenesis, decreased renal ammoniogenesis and H^+^ excretion, and decreased renal reabsorption of filtered ketones. As documented by our case, the term euglycemic ketoacidosis is somewhat misleading, as the major component of the acidosis may be hyperchloremic.

### Increased ketogenesis

SGLT2i lowers blood glucose, leading to decreased insulin secretion and increased glucagon secretion [15]. Similar to starvation, [17] a low insulin-to-glucagon ratio stimulates lipolysis in adipocytes and ketone bodies formation and gluconeogenesis in the liver [15, 18]. However, concomitant stress related precipitants, some form of insulin deficiency or reduced caloric intake are required to promote ketogenesis [18–20]. The two-hit hypothesis proposes that elevated stress-related hormones glucagon, cortisol and catecholamines stimulate ketogenesis in the setting of relative insulin deficiency due to low plasma glucose induced by SGLT2i [18].

### Metabolic renal dysfunction

In the classic DKA, most of the acidosis can typically be explained by pure ketoacidosis with high anion gap [13]. However, hyperchloremia may develop and even predominate in some patients later in the course of DKA, due to urinary loss of ketones and the treatment with normal saline [13, 21]. In contrast, patients with equally severe SGLT2i-associated acidosis had a markedly lower anion gap [19]. Beta-hydroxybutyrate concentration varied substantially in several published cases. In our case, the anion gap was only 18 and the beta-hydroxybutyrate 3.2 mmol/l, despite pH of 6.85 and base excess − 29. Sampani [22] reported a similar case with only a slightly elevated anion gap despite very low pH (Table 1). Compared with DKA, a concomitant metabolic renal dysfunction and slower development of ketosis modify the development of acidosis and enhance the hyperchloremic component. The main renal abnormalities associated with SGLT2i are impaired H^+^ secretion in the proximal tubule, impaired ammoniogenesis and loss of ketoacid salts in the urine.

Impaired H^+^ secretion results from the concomitant inhibition of the H^+^/Na^+^ exchanger (NHE3) in the proximal tubule by SGLT2i [23]. Impaired ammoniogenesis is more complex and is related to excess ATP in tubular cells [16]. Blockade of both SGLT2 and NHE3 reduces the sodium load in the proximal tubule, resulting in reduced ATP consumption by the Na/K-ATPase [16]. In addition, both the fatty acids produced by lipolysis and the ketone bodies filtered in the glomeruli are utilized by renal tubular cells, increasing cellular ATP levels [24].

Since ammonia is the main buffer within the renal tubules, impaired ammoniogenesis hinders further secretion of H^+^ [25]. Finally, the loss of sodium or potassium salts of ketoacids in the urine is equivalent to an indirect loss of bicarbonate, resulting in the normal anion gap acidosis. This is because a molecule of NaHCO3^−^ has been consumed in the formation of ketonate^3^ from the ketoacid [16]. Interestingly, the less impaired ammoniogenesis in the classical DKA [26] allows the excretion of unresorbed ketones in the form of ammonium salts, avoiding any acid-base effects. It should be noted that in the early stages of ketosis, e.g. during the first 4 days of fasting, renal resorption of ketones is saturated at plasma beta-hydroxybutyrate levels of around 2 mmol/l [27].

In short, relative hypoglycemia and stress lead to a decrease in insulin levels and an increase in counterregulatory hormones, resulting in relative insulin deficiency and stimulation of ketogenesis. At the same time, SGLT2i, ketones and fatty acids impair renal H^+^ excretion and ammoniogenesis. Ketones are lost from the kidneys as sodium salts, leading to hyperchloremic acidosis.

### Pathophysiological considerations of the reported case

In our patient, several precipitating factors were present: the major surgery, postoperative fasting and presumably impaired insulin secretion, suggested by the recent addition of insulin to the medication. In addition, the nurses described the patient as very anxious, which may have contributed to increased ketogenesis. In the study of Kubera, [28] even simple psychosocial stress increased the beta-hydroxybutyrate level fourfold.

Rather low level of ketones corresponded to mild DKA [12]. Hyperchloremic acidosis could alternatively be explained by renal tubular acidosis with direct loss of bicarbonate. However, we have found only 3 published cases of severe proximal tubule disorder, Fanconi syndrome, during SGLT2i treatment that presented with renal tubular acidosis with rather low anion gap, [29–31] but all with elevated ketone levels. Due to the anuria of our patient, we could not perform a urine test to refine the diagnosis.

Let us try to assess whether the renal loss of ketones at such a low beta-hydroxybutyrate concentration (3.2 mmol/l) could really have led to such a profound hyperchloremic acidosis within 4 days of surgery. Assume that complete renal resorption of ketones occurs up to the plasma beta-hydroxybutyrate concentration of 2 mmol/l [27]. Correspondingly, the maximum resorption is 300 mmol/day with a normal glomerular filtration rate of 150 l/day [25]. Thus, at a concentration of 3.2 mmol/l, up to approximately 150 mmol of beta-hydroxybutyrate or up to 200 mmol of ketoacids including acetoacetate could be lost per day. If ketones were excreted only as sodium or potassium salts due to impaired ammoniogenesis, up to 200 mmol/day or 800 mmol in 4 days of bicarbonate could be lost indirectly. As shown in Table 1, after correction for increased lactate, the deficit of bicarbonate was approximately 15 mmol/l, corresponding to 200 mmol in 15 l of extracellular fluid, which is far from the theoretically possible 800 mmol, even when intracellular titration is taken into account. Thus, the renal loss of ketones is sufficient to explain the severity of the hyperchloremic acidosis. Note that the patient had already been admitted to the ICU with severe hyperchloremic acidosis without prior treatment with normal saline.

In conclusion, we report a rare case of severe SGLT2i-associated acidosis in the perioperative period in which a rather mild ketoacidosis led to a profound hyperchloremic acidosis. Early diagnosis is of paramount importance for a good patient prognosis. A high index of suspicion is required due to the non-specific symptoms and low glycaemia. According to FDA recommendations, [8] SGLT2i should be discontinued at least 3 days before elective surgery, considering that the elimination half-time is about 12 h and the pharmacodynamic effects may last for several days [32]. This recommendation was not followed in our case, as empagliflozin was continued until the day before surgery. Similarly, consensus guidelines recommend avoiding the use of SGLT2i in case of severe illness and in most cases in-hospital. However, because of the beneficial effects of SGLT2i and the low incidence of eDKA, this widespread policy of discontinuing gliflozin has recently been questioned [33]. However, our case tends to support the appropriateness of this policy.

## Data Availability

Analyses of all data performed during the preparation of the case report are included in this manuscript.

## Footnotes

3 A ketonate is an anion derived from ketoacid.

## Abbreviations

ASA: American Society of Anesthesiologists
ATP: Adenosine triphosphate
CPR: Cardiopulmonary resuscitation
D1: Day 1
DKA: Diabetic ketoacidosis
eDKA: Euglycemic diabetic ketoacidosis
ECG: Electrocardiography
FDA: Food and Drug Administration
HDU: High-dependency unit
ICU: Intensive care unit
NHE3: Proximal H^+^/Na^+^ exchanger
pCO_2_: Partial pressure of carbon dioxide
ROSC: Return of spontaneous circulation
SGLT2: Sodium-Glucose Transport Protein 2
SGLT2i: Sodium-Glucose Transport Protein 2 Inhibitors
T2DM: Type 2 diabetes mellitus
