# Correction: Empagliflozin-associated postoperative mixed metabolic acidosis. Case report and review of pathogenesis

**DOI:** 10.1186/s12902-023-01436-w

**Published:** 2023-08-28

**Authors:** Michal Sitina, Marek Lukes, Vladimir Sramek

**Affiliations:** 1 Department of anesthesiology and intensive care medicine, St. Anne´s University Hospital, Pekarska 664/53, Brno, 656 91 Czech Republic; 2 Department of Biostatistics, International Clinical Research Center, St. Anne´s University Hospital, Pekarska 664/53, Brno, 656 91 Czech Republic; 3 Faculty of Medicine, Masaryk University, Kamenice 5, Brno, 625 00 Czech Republic

**Correction: BMC Endocr Disord 23, 81 (2023).**

10.1186/s12902-023-01339-w

Following publication of the original article [1], the authors reported that the funding information was mistakenly missed in the manuscript.

The correct funding statement should be:

The study was supported by the European Regional Development Fund - Project ENOCH (No. CZ.02.1.01/0.0/0.0/16_019/0000868).

The original article [1] has been updated.
